# Deep Intronic Mutation and Pseudo Exon Activation as a Novel Muscular Hypertrophy Modifier in Cattle

**DOI:** 10.1371/journal.pone.0097399

**Published:** 2014-05-14

**Authors:** Claire Bouyer, Lionel Forestier, Gilles Renand, Ahmad Oulmouden

**Affiliations:** 1 Unité Mixte de Recherche (UMR) 1061 INRA/Université de Limoges, Unité de Génétique Moléculaire Animale, Limoges, France; 2 UMR 1313 INRA, Unité de Génétique Animale et Biologie Intégrative, Jouy-en-Josas, France; Rutgers University -New Jersey Medical School, United States of America

## Abstract

Myostatin is essential for proper regulation of myogenesis, and inactivation of Myostatin results in muscle hypertrophy. Here, we identified an unexpected mutation in the *myostatin* gene which is almost fixed in Blonde d'Aquitaine cattle. In skeletal muscle, the mutant allele was highly expressed leading to an abnormal transcript consisting of a 41-bp inclusion and premature termination codons and to residual levels of a correctly spliced transcript. This expression pattern, caused by a leaky intronic mutation with regard to spliceosome activity and its apparent stability with regard to surveillance mechanisms, could contribute to the moderate muscle hypertrophy in this cattle breed. This finding is of importance for genetic counseling for meat quantity and quality in livestock production and possibly to manipulate *myostatin* pre-mRNA in human muscle diseases.

## Introduction

Myostatin, a member of the transforming growth factor beta (TGF-β) superfamily, functions as a negative regulator of skeletal muscle development and growth. Myostatin is expressed almost exclusively in cells of the skeletal muscle lineage, from the embryonic myotome to striated muscle in adults [1]. The *myostatin* gene has been highly conserved throughout evolution, particularly at the third exon encoding the entire bioactive COOH-terminal in all vertebrate homologs [2], [3]. Like other TGF-β superfamily members, Myostatin is synthesized in a precursor form which dimerizes via disulfide bonds and undergoes three proteolytic cleavages. Removal of the signal peptide sequence is followed by cleavage at a tetrabasic processing site, resulting in a NH2-terminal propeptide and a COOH-terminal peptide. Following this proteolytic processing, the propeptide and disulfide-linked C-terminal dimer remain noncovalently bound in a latent complex. Activation of latent Myostatin can occur by proteolytic cleavage of the propeptide by members of the BMP 1/tolloid family of metalloproteinases, which causes dissociation of the latent complex [4].

Since its discovery in mice in 1997 [1], the *myostatin* gene has been extensively investigated considering the potential benefits of enhancing muscle growth in clinical and agricultural settings. Loss-of-function mutations which impair Myostatin function or those which knockdown *myostatin* gene expression, result in muscle hypertrophy often referred to as “double-muscling” [5]–[10] whereas *myostatin* overexpression induces profound muscle loss [11]. In humans, the first natural *myostatin* mutation has been identified in a young boy [12]. Building on these results, a number of strategies including the use of Myostatin inhibitors or antisense oligonucleotides to manipulate *myostatin* pre-mRNA splicing are being developed for the treatment of muscle-wasting disorders such as Duchenne muscular dystrophy [13], [14].

**Figure 1:**
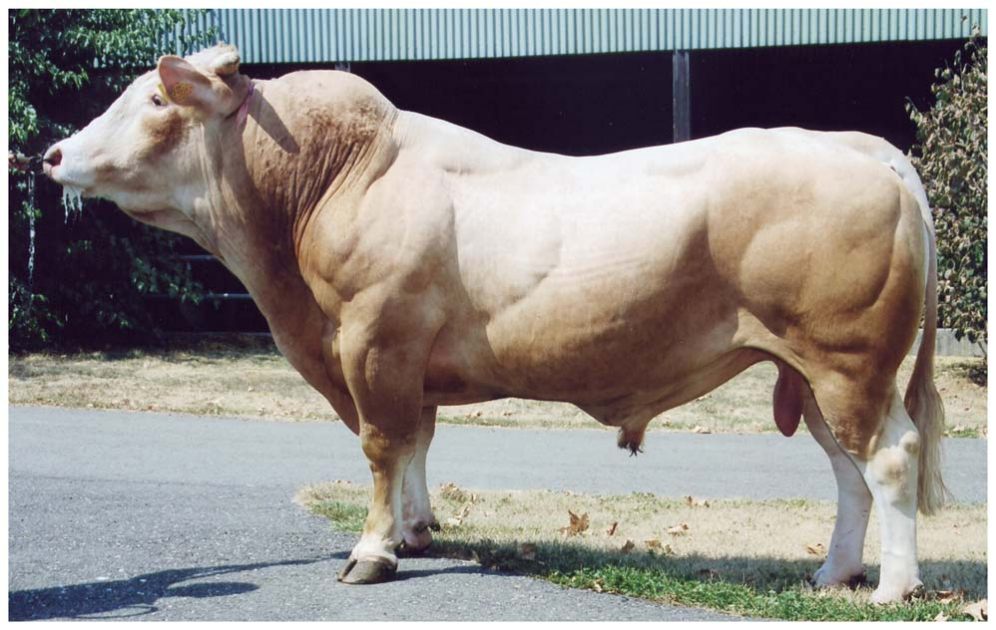
A Blonde d'Aquitaine bull homozygous for the *T3811>G3811* mutation exhibiting muscle hypertrophy.

In cattle, several mutations that cause different degrees of hypermuscularity have been reported. The most extreme phenotype is seen in the Belgian Blue breed (BBB) [7]. The well-muscled French Blonde d'Aquitaine breed (BAB) is renowned for producing high-yielding beef carcasses and displays a less hypertrophic phenotype (Fig. 1) with certain variations in muscle conformation commonly observed between animals. Based on the absence of an altered amino-acid sequence and reduced *myostatin* mRNA levels, the *myostatin* gene does not seem to be responsible for muscle phenotype in BAB [8], [15]. However, these studies did not rule out the existence of an aberrant transcript that may escape surveillance mechanisms.

Here, we identified an unexpected mutation in the *myostatin* gene. In skeletal muscle, the mutant allele was highly expressed leading to an abnormal transcript with a premature termination codon and to residual levels of a correctly spliced transcript.

## Results

### Aberrant *myostatin* mRNA

**Figure 2:**
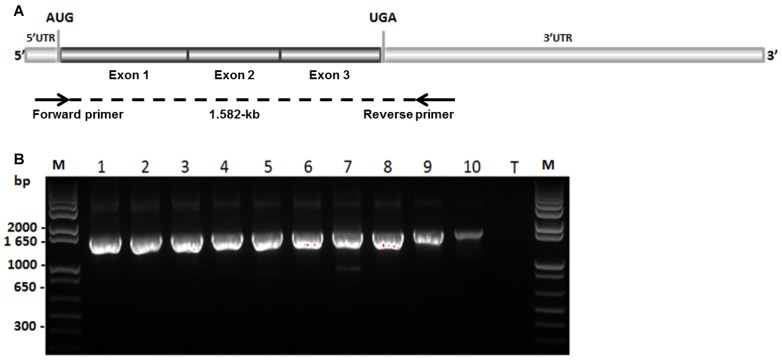
*myostatin* cDNA amplifications. (A) *myostatin* mRNA structure. Arrows show the primers used for PCR amplification. (B) PCR products obtained from *longissimus dorsi* cDNA samples of ten Blonde d'Aquitaine animals (1–10). The predicted size (A) is 1.582-kb. All the amplifications were directly sequenced by the same primers used for PCR amplifications (A). T: PCR assay without sample; M: size marker.

**Figure 3:**
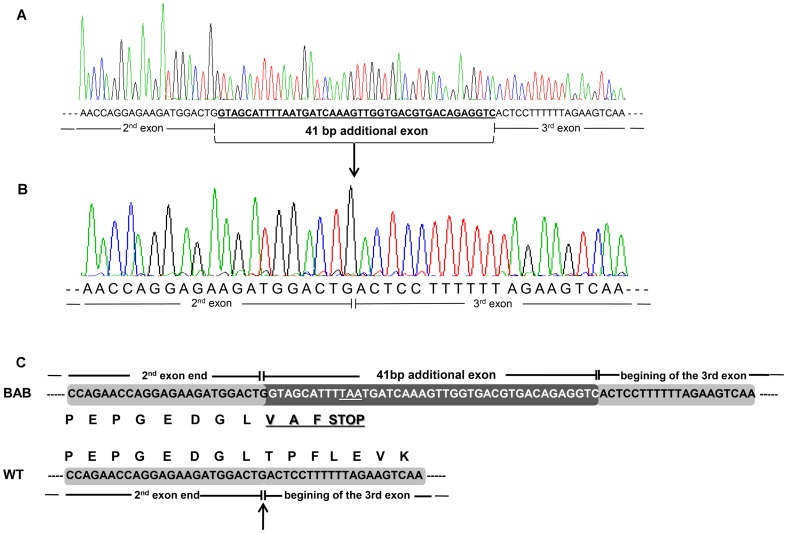
Aberrant *myostatin* cDNA. (A) Nine sequenced Blonde d'Aquitaine (BAB) animals carry a 41-bp additional sequence between the exons 2 and 3. (B) Wild-type *myostatin* cDNA primary structure at the exon2/exon3 junction (WT). (C) The 41-bp extra exon inclusion leads to a premature termination codon. Therefore, translation predicts a truncated protein lacking the entire bioactive region [2], encoded by exon 3. The arrow indicates that the extra exon inclusion occurs at the exon2/exon3 junction of the wild-type transcript. Of note, the premature stop codon occurs 29-bp upstream from the exon2/exon3 junction.

**Figure 4:**
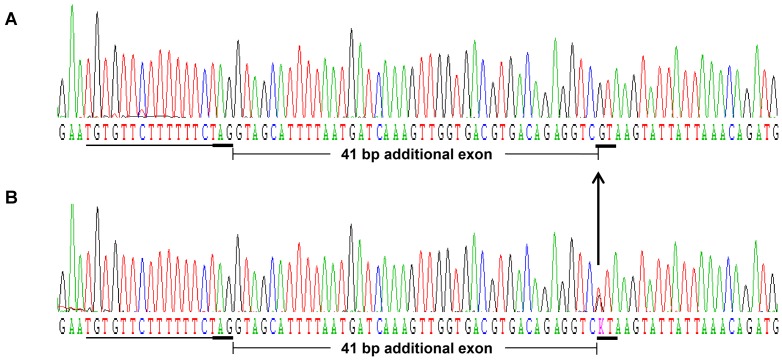
Deep intronic mutation. (A) The *T>G* substitution (arrow) creating a strong 5′ donor splice site (GT, underlined) was found in nine animals at the homozygous (*G/G*) state. (B) The tenth is heterozygous (*T/G*). A perfect preexistent 3′ acceptor splice site: AG and its upstream pyrimidine tract are underlined.

We studied potential transcript abnormalities that could be caused by an intronic mutation. For this, we sequenced the 1.582-kb *myostatin* cDNA obtained by RT-PCR in Blonde d'Aquitaine *longissimus dorsi*, a hypertrophied skeletal muscle in cattle. The amplification products (Fig. 2) encompassed the three coding exons which were 35-bp upstream and 420-bp downstream of the open reading frame respectively. At first glance, these results suggested that BAB animals produce correctly spliced *myostatin* mRNA. Unexpectedly, direct sequencing of these PCR products in 10 fullblood BAB animals revealed a 41-bp insertion between exons 2 and 3 in 9 animals (Fig. 3A, B). The tenth animal exhibited both aberrant and correctly spliced transcripts. Furthermore, this extra exon inclusion contained two successive premature termination codons (PTC). Translation of this aberrant transcript predicts a truncated protein lacking the entire bioactive region [2], [3] encoded by exon 3 (Fig. 3C). Examination of the genomic sequence of the *myostatin* gene showed that the 41-bp insertion was located in intron 2. Closer examination of the flanking sequences showed that this pseudoexon intronic sequence was preceded by a perfect 3′ acceptor splice site (Fig. 4).

### Causative mutation

To identify the cause of this aberrant splicing we carried out genomic DNA sequencing of the entire intron 2 (2.033-kb) in the ten animals. Direct sequencing of PCR products revealed a *T3811>G3811* deep intronic mutation (numbering from the first base of the translation codon at the genomic level). Remarkably, the mutation created a new 5′ donor splice site. Nine animals were found *G/G* homozygous (Fig. 4A) and the tenth was *T/G* heterozygous as expected (Fig. 4B). We did not find any other changes in the remaining intron 2 sequence. These results suggest that the intronic “exonisation” discovered in the BAB animals was caused by a perfect preexistent 3′ acceptor splice site and the *T3811>G3811* mutation. This substitution seems to generate a strong cryptic 5′ donor splice site (Fig. 4). This assumption is supported by the fact that we could not detect any wild-type transcripts by direct cDNA sequencing of all homozygous *G/G* animals (Fig. 3) whereas the heterozygous *G/T* animal exhibited both wild-type and abnormal transcripts.

### Genotyping in different cattle breeds

**Figure 5:**
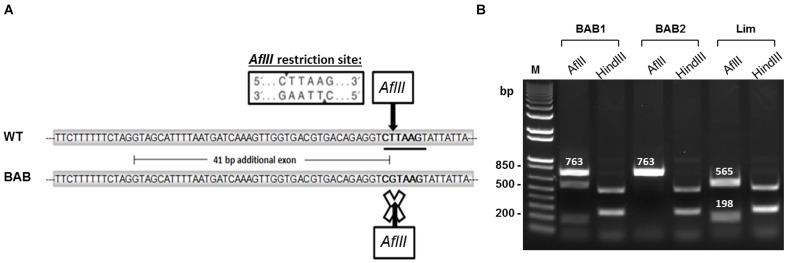
RFLP-PCR genotyping. (A) The *T>G* substitution leads to disappearance of the *AflII* restriction site in Blonde d'Aquitaine (BAB). (B) PCR amplification products from two BAB animals and one Limousine animal (Lim) were digested either by *AflII* or by *HindIII* restriction enzymes. *AflII* digestion differentiates animals which are homozygous *G/G* (BAB2, no digestion, one 763-bp fragment), heterozygous *T/G* (BAB1, three fragments: undigested PCR products corresponding to the mutated allele (763-bp) and PCR products corresponding to the wild-type allele gave two 565-bp and 198-bp fragments) or homozygous *T/T* (Lim, two fragments: 565-bp and 198-bp). *HindIII* digestion was used as a cutting control. WT: wild-type; M: size marker.

We genotyped 445 animals from several European cattle breeds by RFLP-PCR (Fig. 5) for the deep intronic mutation. We found that the mutation was almost fixed in BAB (Table 1); all animals belonging to other cattle breeds were wild-type. Taken together, our findings identify a deep intronic mutation (*T3811>G3811)* leading to an aberrant *myostatin* mRNA. This likely results in a truncated Myostatin, lacking the entire bioactive region and hence reduction in functional Myostatin concentrations contributing to muscular hypertrophy in animals belonging to Blonde d'Aquitaine breed.

**Table 1:** Genotyping of the *T3811>G3811* mutation in European cattle breeds.

Breed/country	Number of animals	*T/T*	*G/G*	*T/G*
Blonde d'Aquitaine/France	60	6	50	4
Limousine/France	42	42	0	0
Aubrac/France	48	48	0	0
Salers/France	33	33	0	0
Maine Anjou/France	20	20	0	0
Parthenese/France	15	15	0	0
Charolaise/France	16	16	0	0
Montbeliarde/France	18	18	0	0
Simmentale/France	9	9	0	0
Prim'Holstein/France	9	9	0	0
Normande/France	10	10	0	0
Gasconne/France	2	2	0	0
Black Japanese/Japan	2	2	0	0
Brave/France	16	16	0	0
Camargue/France	17	17	0	0
Italian brown/Italia	15	15	0	0
Maremmana/Italia	9	9	0	0
Italian red Pezzata rossa/Italia	9	9	0	0
Italian Friesan/Italia	19	19	0	0
Piemontaise/Italia	29	29	0	0
Romagnola/Italia	19	19	0	0
Belgian Blue Breed/Belgium	6	6	0	0
Chianina/Italia	11	11	0	0
Marchigiana/Italia	11	11	0	0
Total	445	391	50	4

### Relative stability of mRNA-containing premature stop codons

The most striking result revealed by our work was the apparent stability of the aberrant transcript despite the presence of premature termination codons as assessed by RT-PCR and direct sequencing (Fig. 2 and Fig. 3). Indeed, eukaryotic mRNAs harboring a PTC are often the targets of nonsense-mediated mRNA decay (NMD) [16].

**Figure 6:**
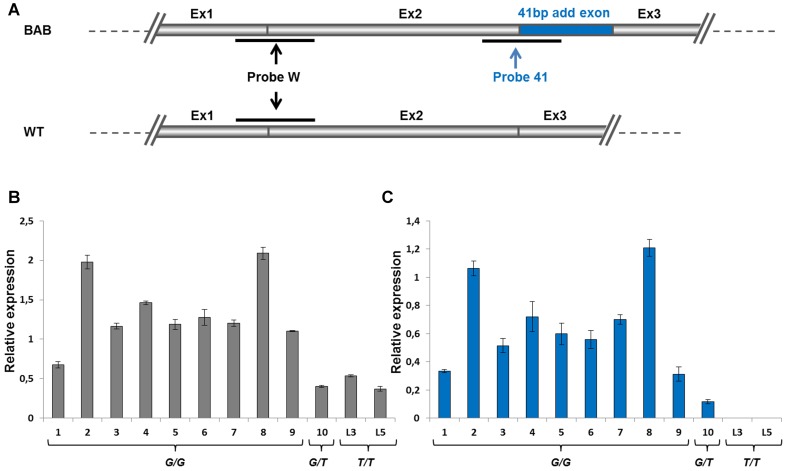
*myostatin* RT-qPCR transcripts. (A) Partial structure of the *myostatin* transcript from Blonde d'Aquitaine (BAB) and wild-type (WT) alleles. At top, the BAB allele contains the 41-bp additional exon (blue) and can be detected on qPCR by the probe 41 (blue) and probe W (black). At bottom, the wild-type allele, without the additional exon can only be detected by the probe W. Relative expression of *myostatin* transcript from twelve muscle samples detected by the probe W (B) and the probe 41 (C). 1-9: BAB homozygous (*G/G*) animals. 10: BAB heterozygous (*G/T*) animal. L3, L5: Limousine homozygous (*T/T)* animals.

To gain more insight into the expression and stability of this aberrant transcript, we developed (Fig. 6A) TaqMan real-time quantitative RT-PCR assays targeted specifically to the 41-bp inclusion (in *G/G* or *G/T* animals) or both to wild-type and abnormal transcripts (in *G/G*, *G/T* or *T/T* animals). As expected, the commonly used TaqMan probe detected *myostatin* mRNA in all animals regardless of their genotype (Fig. 6B). Indeed, the two first exons were shared by abnormal and correctly spliced transcripts (Fig. 6A). The probe targeted to the 41-bp inclusion detected the aberrant transcript only in animals carrying at least one *T3811>G3811* allele (Fig. 6C) as expected. This transcript remained undetectable in the two homozygous (*T/T*) animals used as control (Fig. 6C). Of note, although some fluctuations in expression were seen, both TaqMan probes showed a similar expression pattern in the 10 screened BAB animals (Fig. 6B, C). Interestingly, abnormal transcripts (with a PTC) were more plentiful than wild-type transcripts. Taken together, and although speculative, these results strongly suggest that expressed transcripts harboring PTCs escaped surveillance mechanisms, probably nonsense-mediated mRNA decay (NMD) in this case. One plausible explanation of this NMD resistance is that the PTC is located less than 50 nucleotides from the last exon/exon junction (Fig. 3). In mammals, as a general rule, only a PTC which occurs more than 50 nucleotides upstream from the last exon/exon junction will be subjected to NMD [16]. Otherwise, the increased levels of *myostatin* mRNA in Blonde d'Aquitaine cattle as compared to Limousine cattle (Fig. 6) suggests a negative feedback loop for functional Myostatin (see discussion).

### Residual level of wild-type mRNA

**Figure 7:**
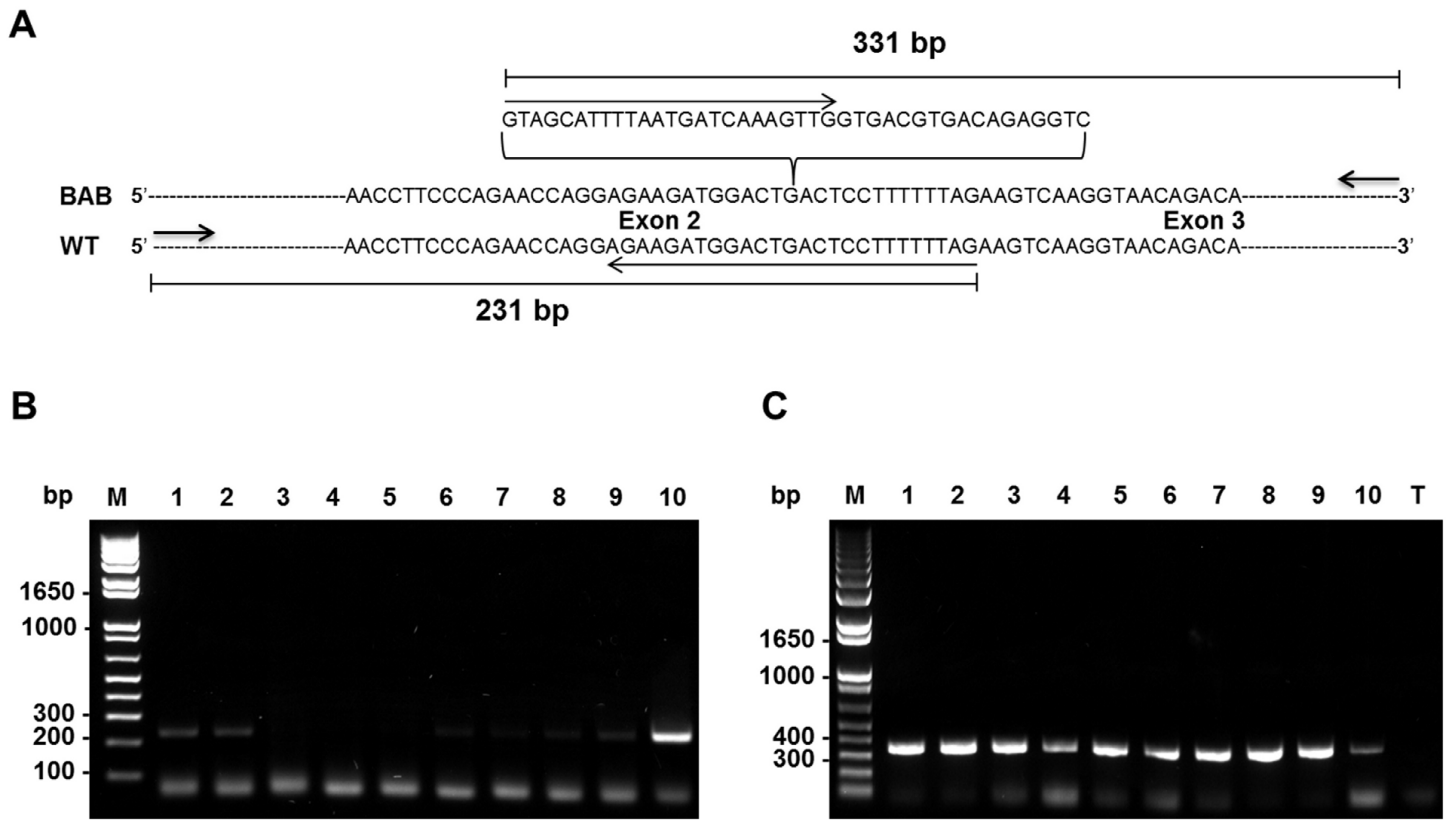
Residual wild-type transcripts. (A) Partial sequence at exon2/exon3 junction of *myostatin* cDNA in Blonde d'Aquitaine (BAB) and normal (WT) cattle. Extra-exonisation in BAB at exon2/exon3 (*G/A*) junction is indicated (bracket). Two specific primers (PCRWT-F/PCRWT-R and PCRBAB-F/PCRBAB-R, Table 2) were used to amplify either the correctly spliced (B) transcript (WT strand) or (C) the transcript with an extra exon (BAB strand). (C) The aberrant transcript was detected at relatively higher levels as compared to the heterozygous *G/T* animal (line 10) in agreement with real-time RT-qPCR data (Fig. 6). Of note, residual correctly spliced transcripts were detected in *G/G* animals at variable levels ranging from undetectable (lines: 3, 4, 5) to detectable levels (lines: 1, 2, 6, 7, 8, 9) as compared to the *G/T* heterozygous animal (line 10). T: PCR assay without sample; M: size marker.

The genotyping studies above showed that the deep intronic mutation was almost fixed in Blonde d'Aquitaine cattle (Table 1). However, some differences in muscle phenotype conformation still exist suggesting that the mutation is leaky with regard to spliceosome activity. Although the wild-type transcript remained undetectable by direct PCR sequencing we performed RT-PCR on cDNA using specific primers to identify residual wild-type transcripts (Fig. 7). Interestingly, this analysis revealed that residual wild-type transcripts, likely resulting from leaky splicing, were apparently produced (Fig. 7B) in homozygous (*G/G*) animals at variable levels ranging from undetectable (lines: 3, 4, 5) to detectable levels (lines: 1, 2, 6, 7, 8, 9) as compared to the *G/T* heterozygous animal (line 10). Aberrant transcripts were more expressed in *G/G* animals than in the *G/T* heterozygous animal (line 10). This result was confirmed by cloning and sequencing. Unfortunately, we were unable to quantify the residual correctly spliced transcript by either semi-quantitative RT-PCR or TaqMan real-time quantitative RT-PCR.

## Discussion

Myostatin is a TGF-β superfamily member that acts as a negative regulator of skeletal muscle growth. Mice lacking Myostatin have a dramatic and widespread increase in skeletal muscle mass as a result of a combination of muscle fiber hypertrophy and hyperplasia [1]. Naturally occurring mutations in the *myostatin* gene also lead to hypermuscular phenotype in cattle breeds such as the Belgian Blue and Piedmontese [5]–[7]. Blonde d'Aquitaine cattle have a less hypertrophic phenotype (Fig. 1) with certain variations in muscle conformation commonly observed between animals.

Here, we have shown that *T3811>G3811* mutation within intron 2 of the *myostatin* gene (Fig. 4) is almost fixed in Blonde d'Aquitaine cattle (Table 1). This is a deep intronic mutation which creates an illegitimate strong cryptic 5′ donor splice site (Fig. 4). In skeletal muscle, the mutant allele was highly expressed (Fig. 6 and Fig. 7C) leading to an abnormal transcript harboring a premature termination codon (Fig. 3C) and to residual levels of a correctly spliced transcript (Fig. 7B), likely resulting from leaky splicing. Translation of the aberrant transcript predicts a non-functional protein lacking the entire bioactive region [2], [3] (Fig. 3C). This prediction is supported by the fact that *myostatin* gene is up-regulated (Fig. 6) in Blonde d'Aquitaine (see below). On the other hand, the residual correctly spliced transcript detected by RT-PCR (Fig. 7B), predicts that some functional Myostatin is also produced. Unfortunately, we were unable to quantify this residual correctly spliced transcript by both semi-quantitative RT-PCR and TaqMan real-time quantitative RT-PCR. However, although speculative, this expression profile might result in reduction in functional Myostatin concentrations and hence the moderate hypertrophic phenotypes of Blonde d'Aquitaine cattle.

Like other TGF-β superfamily members, the mature Myostatin signals via the activin type II receptors (ActRIIA and ActRIIB) and activin type I receptors (ALK4 and ALK5) to phosphorylate responsive Smad proteins (Smad2 and Smad3, Smad2/3), which enables the Smad proteins to form a transcriptional complex with Smad4 to transcribe target genes [17]. Myostatin activity is controlled at various levels by different mechanisms, including a range of extracellular antagonists that interact with Myostatin and prevent receptors activation [3], [18], [19]. Furthermore, several reports have shown that *myostatin* gene expression is regulated at the transcriptional level in humans and cattle through *cis* regulatory elements and *trans* acting factors [20], [21]. Otherwise, expression studies revealed that there is increased expression of *myostatin* mRNA in the muscle of “double-muscled” cattle that carry a loss-of-function mutation in *myostatin* gene when compared to the expression in normal muscled cattle [22]–[24]. Based on these results, it has been suggested that Myostatin could regulate the expression of its own gene. Subsequently, it was confirmed that Myostatin indeed auto-regulates its own expression by feedback loop via Smad7, a potent inhibitor of signaling by TGF-β and activins [25]. Of note, this study has shown that Belgian Blue cattle that express non-functional *myostatin* allele produce relatively higher quantities of *myostatin* mRNA in the *biceps femoris* muscle as compared to the normal cattle [25]. Our expression analysis is consistent with those data (Fig. 6 and Fig. 7). Virtually all *G/G* homozygous animals exhibit relatively higher levels of *myostatin* transcripts in the *longissimus dorsi* muscle as compared to both *G/T* heterozygous and *T/T* wild-type animals. Thus, up-regulation of the *myostatin* gene in *G/G* Blonde d'Aquitaine animals suggests that the negative feedback loop regulated by functional Myostatin is altered in this breed. Although it remains to be proven, these expression analyses suggest that non-functional Myostatin is produced in Blonde d'Aquitaine animals and is correlated with high levels of the aberrant transcript. In this regard, it will be of a great interest to compare this auto-regulation in Belgian Blue animals where a functional Myostatin protein is almost certainly not produced and in Blonde d'Aquitaine animals with a leaky mutation which probably produces some functional Myostatin from residual correctly spliced mRNA. This is all the more interesting since truncated Myostatin from Blonde d'Aquitaine cattle lacks the entire bioactive domain whereas that of the Belgian Blue breed exhibit a portion of the bioactive domain. Furthermore *myostatin* mRNA-containing PTCs that lead to each truncated Myostatin [5]–[7] seem to have escaped surveillance mechanisms, most likely nonsense-mediated mRNA decay (NMD) in this case. In Blonde d'Aquitaine and Belgian Blue cattle, the PTC is located less than 50 nucleotides from the last exon/exon junction (Fig. 3) and in the last exon (that is not followed by an exon–exon junction) respectively. In mammals, as a general rule, only a PTC, which occurs at more than 50 nucleotides upstream from the last exon/exon junction, will be subjected to NMD [16].

In conclusion, we identified a mutation in the *myostatin* gene that most likely contributes to muscular hypertrophy of the Blonde d'Aquitaine breed. Taken together, it is attempting to speculate that this leaky mutation, with regard to spliceosome activity, appears to buffer the nonsense mutation and prevents extreme muscle overgrowth making it biologically significant in the context of mutations causing hypermuscularity. Indeed, extreme muscle overgrowth in cattle has disadvantages including reduction in female fertility, lower viability of offspring, delay in sexual maturation and systematic use of cesarean delivery due to large calf size. This finding is of importance for genetic counseling for meat quantity and quality in livestock production and possibly opens a new therapeutic option using *myostatin* pre-mRNA to treat human muscle diseases.

## Materials and Methods

### Blonde d'Aquitaine animals and muscle samples

Ten 12 months old Blonde d'Aquitaine bulls were used in this study. They were reared in the same conditions of feeding, housing and health status at the beef progeny test station of MIDATEST (Pyrénées Atlantiques, France). *Longissimus dorsi* samples were collected within 15 minutes after slaughter (slaughterhouse of Pau, France), cut into small pieces and immediately frozen in liquid nitrogen, transported to the laboratory and stored at −80°C until use. Muscle samples (used as controls) from two animals belonging to the Limousine breed were obtained from Limoges slaughterhouse (France).

### DNA and RNA extractions

DNA was extracted from the ten muscle samples using the QIAamp DNA midi kit (Qiagen) according to the manufacturer's instructions and stored at −20°C. Total RNA was extracted from muscle samples using Tri reagent (Sigma-Aldrich) and treated with DNase I (Sigma-Aldrich) following manufacturer's instructions. The quality and quantity of RNA were evaluated by the Agilent 2000 bioanalyzer and conserved at −80°C until use.

### RT-PCR and quantitative real-time RT-PCR

RT-PCR and quantitative real-time RT-PCR assays for RNA analysis were performed to detect an aberrant *myostatin* transcript (RT-PCR assay) that may escape surveillance mechanisms (quantitative real-time RT-PCR). 2 µg of total RNA from the ten Blonde d'Aquitaine bulls and two Limousine animals were reverse-transcribed (RT) into cDNA using the high-capacity cDNA reverse transcription kit (Applied Biosystems). 100 ng (RNA equivalents) of cDNA for each sample were used as template (RT-PCR assay) to amplify the entire coding region of the *myostatin* transcript (Fig. 2A) or to detect residual wild-type transcripts (Fig. 7). PCR amplifications were carried out in a 25 µL reaction volume containing 10 pmol of each primer (Table 2) and 12.5 µL of 2X working concentration PCR Master Mix (ABgene, Thermo Scientific) with the following cycling conditions: initial denaturation at 95°C for 2 min followed by 35 cycles (95°C for 30 s, 55°C for 30 s, 72°C for 2 min) and one cycle (72°C for 5 min). PCR products (Fig. 2B) were purified with 1 µl ExoSAP-IT (USB products, Affymetrix) per 5 µl of PCR product, and then incubated 60 min at 37°C and 15 min at 80°C. Purified PCR products were sequenced on both strands using the same primers and the BigDye Terminator v1.1 Cycle Sequencing Kit (Applied Biosystems) according to manufacturer's instructions.

**Table 2:** List of primers used in this study.

Name	Sequence (5′-3′)	Template	Objectives
Intron2-F	ACTGTCTTACTGTTCTTTAACAGGAG	Genomic DNA	RFLP-PCR (Fig. 5)
Intron2-R	GAGAGGCACAGACTCAGAAGAAGATA		
PCRWT-F	ATCAAACCCATGAAAGACGGTACAAG	cDNA	Wild-type RT-PCR (Fig. 7B)
PCRWT-R	AGAAGATGGACTGACTCCTTTTTTAG		
PCRBAB-F	GTAGCATTTTAATGATCAAAGTTG	cDNA	BAB RT-PCR (Fig. 7C)
PCRBAB-R	TGCTGTACTCCTACAAAGATGTCT		
Intron2seq-F	ATGTGAAGACAGTGTTGCAGAACTGGCTCA	Genomic DNA	Entire intron 2 amplification
Intron2seq-R	TCTGTGGAGTGTTCATCACAATCAAGCCCA		
5′UTR-F-2	AGAACAAGGGAAAAGATTGTATTGATTTTA	cDNA	RT-PCR (Fig. 2)
3′UTR-R-2	AAATAATGGTATATAACAATACTGCA		

Quantitative real-time RT-PCR assays were performed in triplicate for each sample using 100 ng cDNAs prepared as described above. Relative amounts either of wild-type and aberrant *myostatin* transcripts or only aberrant transcripts were identified by the available Taqman probe (Bt03217979_m1) and a specific TaqMan probe (custom design service, Applied Biosystems) respectively. The positions of TaqMan probe hybridization targets are indicated in Figure 6. Two genes, *CASC3* (*cancer susceptibility candidate 3*, TaqMan probe: Bt0326991_m1) and *SF3A1* (*splicing factor 3a, subunit 1*, TaqMan probe: Bt03254301_m1) were used as internal control for more accurate normalization of expression data. cDNAs were amplified on an ABI PRISM©7900 system (Applied Biosystems) according to manufacturer's instructions. Relative mRNA expression values were calculated by the ΔΔCt method with normalization of each sample to the average change in cycle thresholds of controls.

### *myostatin* intron 2 sequencing

The 2.033-kb of *myostatin* intron 2 was amplified from DNA from the ten Blonde d'Aquitaine animals using Taq DNApolymerase Expand Long Template (Roche Applied Science) according to manufacturer's instructions; intron2seq-F and intron2seq-R primers used are indicated in Table 2. PCR products were purified and sequenced on both strands as described above.

### RFLP-PCR assays

We genotyped 445 animals from several European cattle breeds (Table 1) by RFLP-PCR (Fig. 5) for the deep intronic mutation (*T3811>G3811*). A 763-bp fragment encompassing the SNP was amplified by PCR from genomic DNA using Intron2-F and Intron2-R primers (Table 2), digested using *AflII* restriction enzyme (Biolabs) and size fractionated by agarose gel electrophoresis (Fig. 5). The *T3811>G3811* SNP destroys a restriction site that cleaves the wild-type *T3811* allele (but not the mutated *G3811* allele) into 565-bp and 198-bp fragments (Fig. 5). Digestion by *HindIII* (a unique site shared between both alleles) was used as a cutting control.

Digestions with *AflII* and *HindIII* were made using 10 µl unpurified PCR products, 2 µl enzyme buffer and 1 µl restriction enzyme in 20 µl final volume. 0,2 µl BSA were added for the *AflII* digestion. Samples were incubated overnight at 37°C.
